# Draft Genome Sequence of *Streptomyces* sp. Strain FB2, Isolated from Rice Rhizosphere

**DOI:** 10.1128/mra.00090-22

**Published:** 2022-05-09

**Authors:** Dhivya Priya Thenappan, Luz Ángela González-Salazar, Cuauhtémoc Licona-Cassani, Annapurna Kannepalli

**Affiliations:** a Division of Microbiology, ICAR-Indian Agricultural Research Institute, New Delhi, India; b Industrial Genomics Laboratory, School of Engineering and Sciences, Tecnológico de Monterrey, Monterrey, Mexico; SIPBS, University of Strathclyde

## Abstract

*Streptomyces* sp. strain FB2 is an actinomycete isolated from rice rhizosphere. A whole-genome assembly of the strain FB2 comprised 7,727,571 bp (number of contigs, 55; GC content, 71.96%). In total, 17 biosynthetic gene clusters (BGCs), including nonribosomal peptides, polyketides, terpenes, and ribosomally synthesized and posttranslationally modified peptides, were predicted.

## ANNOUNCEMENT

*Streptomyces*, the largest genus (more than 500 species) of phylum *Actinobacteria*, is well known for the production of bioactive molecules which effectively control various plant diseases via two distinct mechanisms of antibiosis and induced resistance (1, 2). In order to explore its biosynthetic potential, we sequenced the genome of *Streptomyces* sp. strain FB2.

*Streptomyces* sp. strain FB2 (MTCC 13139) was isolated from rice rhizosphere in Telangana, India (17.32°N, 78.39°E). Briefly, 1 g of soil attached tightly to the roots was collected, suspended to prepare serial dilutions, and plated onto ISP2 agar plates. After 5 days of incubation at 30°C, a small colony with a powdery appearance was selected, and an axenic culture was obtained by streaking it onto fresh plates.

The genomic DNA was extracted by suspending 5-day-old pure colony in the buffer of FastDNA spin kit for soil (MP Biomedicals, Santa Ana, CA) and following the manufacturer’s protocol. For primary identification, the 16S rRNA was amplified using universal primers (27F and 1522R). The PCR reaction (25 μL) consisted of 1× Go *Taq* buffer, 0.25 μM each primer,1 μL DNA template, 0.2 mM each dNTPs, 2 mM MgCl_2_, and 1.25 U *Taq* polymerase. The reaction conditions were as follows: 95°C for 5 min; 25 cycles of 94°C for 60 s, 60°C for 30 s, and 72°C for 90 s; and a final extension at 72°C for 7 min. The amplicon sequenced and compared against the EzBioCloud database (7 July 2021 update) revealed that *Streptomyces* sp. FB2 (GenBank accession number MZ736626) is closely related to Streptomyces longispororuber (GenBank accession number AB184440) with 98.66% sequence identity (3). We further sequenced *Streptomyces* sp. FB2 genomic DNA using the Illumina NextSeq 500 (2 × 150 bp) platform. Paired-end sequencing libraries prepared using TruSeq Nano DNA library kit generated 17,938,298 reads which were trimmed using a sliding window quality cutoff of Q15 with Trimmomatic (v 0.36) (4) and quality assessed with FastQC (v 0.11.5) (5). Filtered reads were assembled with SPAdes v 3.11.1 (6). The genome of *Streptomyces* sp. FB2 consists of 7,727,571 bp (55 contigs; *N*_50_, 390,822 bp) assembled into 52 scaffolds. After genome assembly, a full genomic coverage at a 240× sequencing depth was obtained. The genome was annotated with the NCBI Prokaryotic Genome Annotation Pipeline (PGAP v 5.3) (7) that identified 7,014 total coding DNA sequences (CDS), 6,840 protein-coding genes, 80 rRNAs (3 5S rRNAs, 4 16S rRNAs, and 2 23S rRNAs), 68 tRNAs, 3 noncoding RNAs (ncRNAs), and 172 pseudogenes.

**FIG 1:**
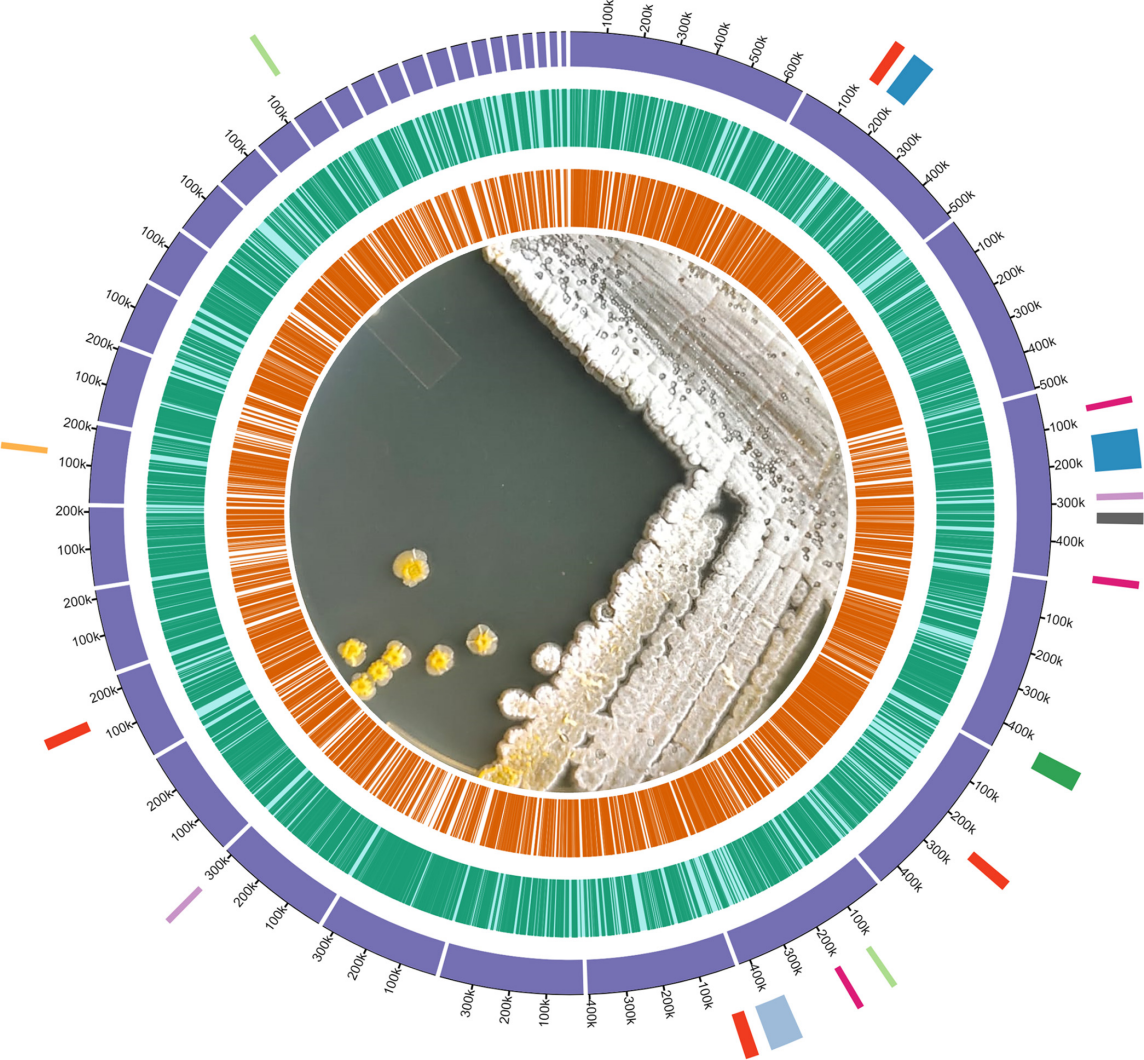
Graphical representation of the *Streptomyces* sp. FB2 genome. From the center to the outside ring the following is shown: genes on the reverse strand (orange), genes on the forward strand (blue), scaffolds (violet), and biosynthetic gene clusters colored by chemical family (red, terpene; blue, nonribosomal peptide synthetase [NRPS]; pink, siderophore; lilac, ribosomally synthesized and postranslationally modified peptides [RiPPs]; gray, butyrolactone; dark green, T3PKS; light green, melanin; light blue, T2PKS; orange, ectoine). In the center is shown the macroscopic morphology of strain FB2 on ISP2 medium with yellow substrate mycelium and white spore mass.

The biosynthetic gene cluster (BGC) repertoire potential in strain FB2 was screened using antiSMASH v 6.0 (8) and BIGFAM v 1.0.0 (9) to evaluate BGC completeness. Default parameters were used for all the software unless otherwise specified. A graphical representation (using the software CIRCA v1.0, http://omgenomics.com/circa/) of the genome and BGC annotation are shown in Fig. 1. A further analysis of the metabolites encoded by these BGCs could be relevant for finding novel bioactivities of industrial and agricultural interest.

### Data availability.

The draft genome sequence of *Streptomyces* sp. FB2 has been deposited in the DDBJ/ENA/GenBank database under the accession number JAKFQV000000000 (BioProject number PRJNA756257, BioSample number SAMN20862081, and SRA accession number SRX13797850).
